# Physical and Chemical Properties of Some Imported Woods and their Degradation by Termites

**DOI:** 10.1673/031.013.6301

**Published:** 2013-06-25

**Authors:** Rashmi R. Shanbhag, R. Sundararaj

**Affiliations:** Institute of Wood Science and Technology, 18^th^ cross, Malleswaram, Bangalore, 560003

**Keywords:** cellulose, density, lignin, total phenolic, wood resistance

## Abstract

The influence of physical and chemical properties of 20 species of imported wood on degradation of the wood by termites under field conditions was studied. The wood species studied were: Sycamore maple, *Acer pseudoplatanus* L. (Sapindales: Sapindaceae) (from two countries), Camphor, *Dryobalanops aromatic* C.F.Gaertner (Malvales: Dipterocarpaceae), Beech, *Fagus grandifolia* Ehrhart (Fagales: Fagaceae), *F. sylvatica* L. (from two countries), Oak, *Quercus robur* L., Ash, *Fraxinus angustifolia* Vahl (Lamiales: Oleaceae), *F. excelsior* L., Padauk, *Pterocarpus soyauxii* Taubert (Fabales: Fabaceae), (from two countries), Jamba, *Xylia dolabrifiormis* Roxburgh, *Shorea laevis* Ridley (Malvales: Dipterocarpaceae), *S. macoptera* Dyer, *S. robusta* Roth, Teak, *Tectona grandis* L.f. (Lamiales: Lamiaceae) (from five countries), and rubber tree, *Hevea brasiliensis* Müller Argoviensis (Malpighiales: Euphorbiaceae) from India. The termites present were: *Odontotermes horni* (Wasmann) (Isoptera: Termitidae), *O. feae, O. wallonensis,* and *O. obeus* (Rambur). A significant conelation was found between density, cellulose, lignin, and total phenolic contents of the wood and degradation by termites. The higher the density of the wood, the lower the degradation. Similarly, higher amount of lignin and total phenolic contents ensured higher resistance, whereas cellulose drives the termites towards the wood.

## Introduction

Wood is susceptible to biodegradation by a variety of organisms. Protecting wood from biodegration and the resulting economic losses is a major challenge. Among the biodeteriorating organisms, termites are a major threat to the service life of wood as they mainly feed on cellulose (Scheffrahn 1991). Factors affecting wood consumption by termites are numerous and complexly related. Among the most important of these factors are wood species, hardness, presence of toxic substances, feeding inhibitors or deterrents, and moisture content of the wood and soil (Smythe et al. 1971; Carter and Smythe 1974). The physical, mechanical, and chemical properties of wood are probably interdependent and affect the wood's resistance to termites. Among the physical factors, wood density influences the termite's ability to fragment the wood mechanically with its mandibles (Bultman et al. 1979), whereas the moisture content drives the termite towards the wood. Wood is a complex, heterogenouse aggregate of cell wall fibers composed primarily of cellulose and hemicellulose, joined by polymers of lignin, to form rigid lignocellulosic matrix, and symbiont and enzyme degraded cellulosic polysaccharides provide the principal carbohydrate component in the diets of wood feeding termites (La Fage and Nutting 1978). Lignin and other chemical extractives, such as alkaloids, phenols, resins, terpenes, essential oils, quinones, silica etc., appear to play the most vital role in preventing the degradation of wood by termites (Oshima 1919; Bavendumm 1955; Walcott 1957; Sandermann and Dietrichs 1957; Sen Sarma et al. 1975; Gupta and Sen Sarma 1978). They act as toxicants, feeding deterrents, repellants, or as non-preferred substrates (Walcott 1946; Abushama and Abdel Nur 1973; Scheffrahn 1991). The present study aimed to relate the physical and chemical properties of different kinds of wood commonly imported to India with degradation by termites in natural condition.

## Materials and Methods

A field experiment was conducted on twenty species of wood, *Acer pseudoplatanus, Dryobalanops aromatica, Fagus grandifolia, Fa. sylvatica, Fraxinus angustifolia, Fr. excelsior, Pterocarpus soyauxii, Quercus robur, Shorea laevis, S. marcoptera, S. robusta, Tectona grandis,* and *Xylia dolabriformis,* imported from different countries, and a native grown rubber, *Hevea brasiliensis,* owing to its well-known susceptibility to termite attack (Table 1). Ten stakes measuring 30.5 cm × 3.8 cm × 3.8 cm and consisting of pure heartwood, avoiding sapwood and pith, of each species of wood were prepared. Care was taken to ensure that the stakes selected belonged to different logs in order to avoid pseudo-replications, and were free from large knots, stains, moulds, decay, and other defects. The stakes were dried to attain constant weight and then labelled. The stakes were weighed and then implanted in soil 60 cm apart in a completely randomized design at a termite test yard, which was severally infested with termites. The test yard was located in Nallal, Karnataka, India, between 13° 4′ 0″ N and 77° 47′ 53″ E, which has a semi-arid climate, an average rainfall of 826 mm, and a temperature range between 18.8 and 29.3° C. The soil is red, loamy, and acidic, with a water holding capacity of 37.77 mm per 15 cm of soil depth. This area is prone to termite attack, and has no fungal activity because of its dry weather. Each stake was half buried, for exposure to termite degradation in the ground condition. After a period of six months, the stakes were removed, brought back to laboratory, rinsed and scrubbed with a brush to remove all soil and carton material, and then oven dried and weighed. The weight loss was calculated by subtracting the weight of the stake recovered from the initial weight of the wood stake. The termites active in the area and on the test stakes were collected, preserved in 70% ethanol, and identified using taxonomic keys. Simultaneously, all the 21 wood species used in study were analyzed for their density (Indian standard 401:^1982^), cellulose content by Anthrone reagent method (Sadasivam and Manickam 1992), lignin content by Klason lignin method (Rowell 2005), and total phenolic content by Folin-Ciocalteau reagent method (Sadasivam and Manickam 1992).

**Figure 1.**
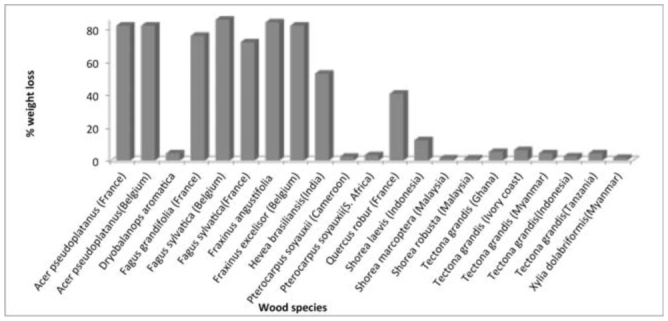
Number of individuals of the four termite species found. High quality figures are available online.

**Figure 2.**
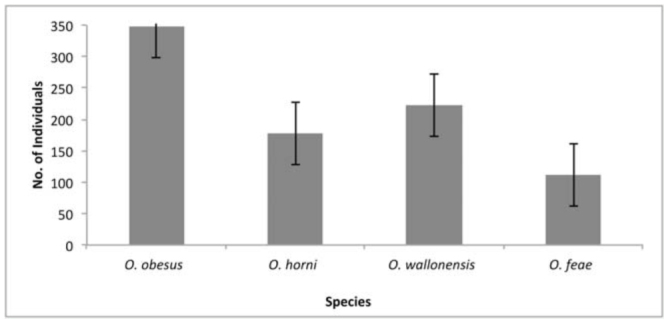
Percentage of weight loss for each wood species. High quality figures are available online.

### Statistical Analysis

From the data collected, mean weight loss due to termite consumption was calculated. Differences in wood consumption by termites were compared using one-way Kruskal Wallis ANOVA because the test for normality failed. Mean weight loss was correlated with the physical and chemical parameters. All statistical analysis was performed by using SigmaStat® 3.1 statistical software (Systat, www.systat.com).

## Results and Discussion

The test yard was found infested with four species of termites, namely *Odontotermes horni* (Wasmann) (Isoptera: Termitidae), *O. feae, O. wallonensis,* and *O. obeus* (Rambur). *O. obesus* was dominant among the species, followed by *O. feae, O. horni,* and *O. wallonensis* (Figure 1). The test yard is known for severe termite infestation, and the present observation confirms earlier reports (Krishnan and Sivaramakrishnan 1993; Rajamuthukrishnan et al. 2004). No fungal activity was observed on the wood species in the test yard, most likely due to the semi arid nature of the area, which has low atmospheric moisture and a scarcity of water. Therefore, the mass loss of wood in the experimental layout was found to be exclusively due to termite degradation, with different levels of weight loss in six months of implantation. The result of the Kruskal-Wallis one-way ANOVA on ranks revealed that there was a statistically significant difference (H = 101.017, df = 20, *p* = 0.001) in the rate of degradation in different wood species. *Fa. sylvatica, Fr. angustifolia, Fr. excelsior, A. pseudoplatanu,* and *H. brasiliensis* species experienced the most degradation. *Q. robur* showed moderate levels of degradation (Figure 2). The other species did not show any sign of termite attack.

Resistance is a critical determinant of life span of tree species. Many heartwood species are known for their resistance against degradation (Harris 1961). Tree species such as *T. grandis* (Oshima 1919; Bavendumm 1955; Sandermann and Dietrichs 1957; Walcott 1957; Behr et al. 1972)), *S. robosta* (Sen Sarma et al. 1975), *Shorea* sp. (Mohd Dahlan and Tarn 1985), *S. marcoptera, Dryobalanops* sp., and *Xylia dolabriformis* (Ling 1996; Grace et al. 1998; Wong et al. 2005), and *Pterocarpus soyauxii* (Nzokou et al. 2005) are well-known for their durability from ancient times. In our study, *Q. robur* was found to be moderately resistant against degradation under Indian conditions. Rapp and Augusta (2006) considered *Q. robur* as a less durable wood species as per European standards. In our study, *Fa. grandifolia, Fa. sylvatica, Fr. angustifolia, Fr. excelsior, A. pseudoplatanus,* and *H. brasiliensis* were found to be susceptible woods. These results are in corroboration with the findings of Badawi et al. (1985) and Evans et al. (2008).

**Figure 3.**
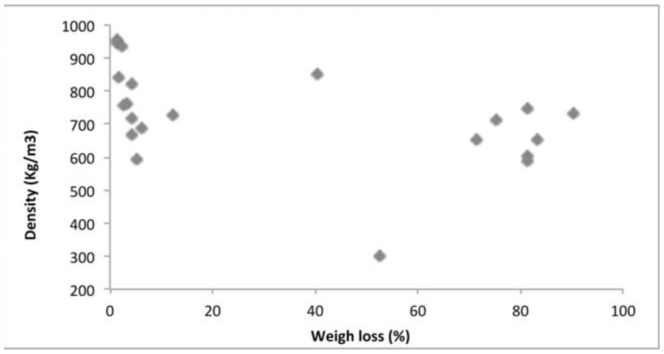
Relationship between density and weight loss in wood. High quality figures are available online.

**Figure 4.**
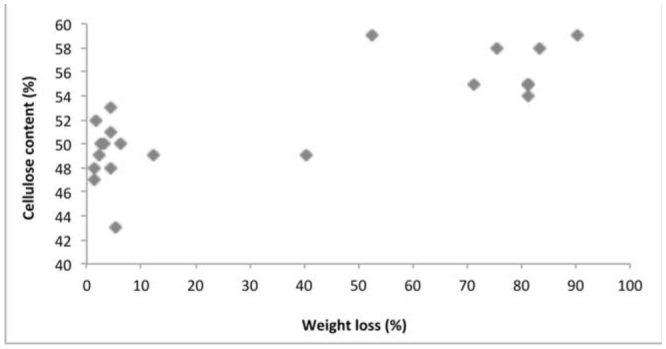
Relationship between cellulose content and weight loss in wood. High quality figures are available online.

**Figure 5.**
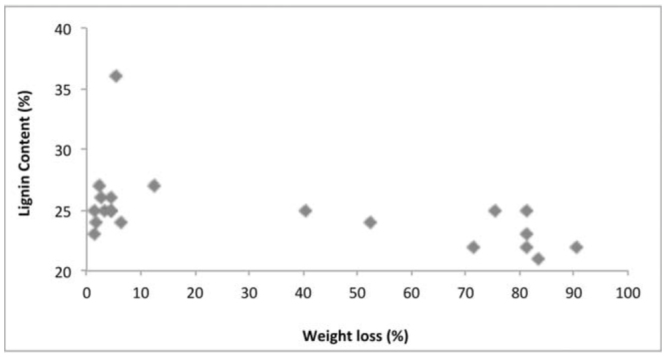
Relationship between lignin content and weight loss in wood. High quality figures are available online.

**Figure 6.**
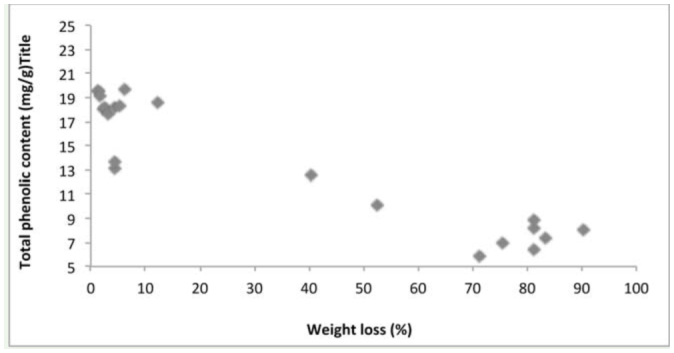
Relationship between total phenolic content and weight loss of tree species. High quality figures are available online.

Several physical and chemical factors determine natural resistance of wood against termite attack. The density of the tested wood species ranged from 0.589 (*Fr. excelsior* from Belgium) to 0.955 g/m^3^ (*S. robusta* from Malaysia). Chemical analysis of imported wood species indicated cellulose content ranged from 43% in *T. grandis* from Myanmar to 59% in *S. marcoptera* from Malaysia. Lignin content was highest in *T. grandis* from Myanmar (36%) and lowest in *Fa. sylvatica* and *A. pseudoplatanus* from France (22%). The total phenolics varied between 271 mg/100g in *Fa. sylvatica* from France to 768 mg/100g in *S. laevis* from Indonesia. The correlation matrix (Table 2) between physical and chemical properties of wood and weight loss due to termite attack showed that the resistanceability of the wood was significantly associated to its physical and chemical properties. Correlation analysis between the chemical composition of the imported wood species and the weight loss percentage due to termite attack indicated that the density (Figure 3), cellulose (Figure 4), lignin (Figure 5), and total phenolic contents (Figure 6) were significantly conelated with degradation by termites at a 5% level of significance.

Density had the greatest effect on the rate of degradation, and it was found that the higher the density of the wood, the more the wood was able to resist termites. A similar relationship was established by Da Costa and Osborne (1967) and Takahashi and Krishima (1973). Behr et al. (1972) reported a negative correlation between wood density and consumption by termites. Bultman and Southwell (1976), Bultman et. al. (1979), and Abreu and Silva (2000) stated that termites prefer less dense woods, due to the ease of mechanically breaking down the wood. Bultman and Southwell (1976) studied the natural resistance of the wood of 114 arboreal species in the forests of Panama and concluded that density is one of the factors that makes wood resistant to termites; the denser and heavier woods presented greater natural durability and resistance to infestation by subtenanean termites.

It is important to highlight that wood density is not the only factor providing resistance to termites. According to Bultman and Southwell (1976), Moore (1979), and Barbosa et al. (2003), the high resistance of some wood species to termites can be explained by the properties of their chemical components, because there is a close relationship between the percentage of toxic extracts and the natural durability of the wood. Tsunoda (1990) concluded that chemical compositions of wood extracts are valuable for understanding resistance to biodegradation.

Chemical constituents, such as cellulose, lignin, and total phenolic content, of wood influenced the rate of degradation, and it was found that the higher the cellulose content, the higher the susceptibility to termite attacks. However, the higher the lignin and total phenolic content, the higher the resistance of wood species. Cellulose is one of the factors that drive termites towards wood species, as it is a primary food source for termites (La Fage and Nutting 1978), which explains the significant positive correlation of cellulose content and wood degradation. Lignin acts as a physical barrier, which is unpalatable to termites (Walcott 1946; Syafii et al. 1988). Extractives and other phenolic compounds of wood also impart higher resistance to termite attack. Wood species with lower amounts of cellulose and higher amounts of lignin and total phenol were termite resistant, while the wood with higher amounts of cellulose and lower amounts of lignin and total phenol were susceptible to termite damage. Previous studies correlating resistance of wood and higher amounts of lignin and extractives in *T. grandis* (Sen Sarma et al. 1975) and in *Eusideroxylon zwageri* and *Neobalanocarpus heimi* (Syafii et al. 1988) are in concordance with the present study.

**Table 1.**
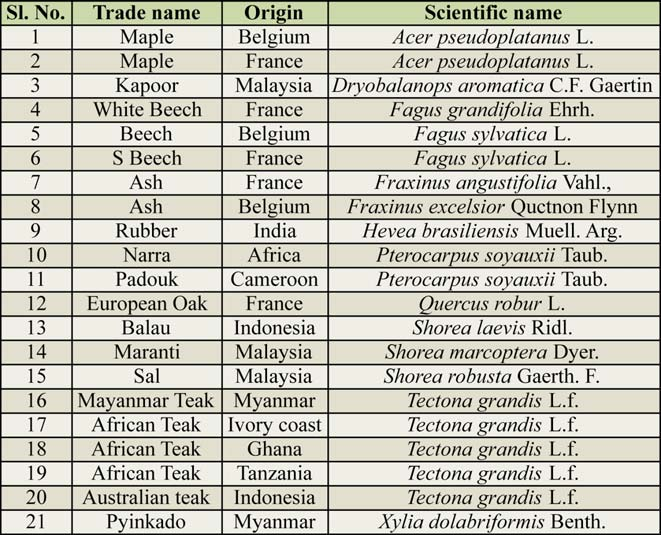
Details of the wood species used in this study.

**Table 2.**
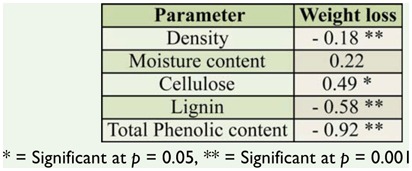
Correlation matrix for the rate of degradation with physical and chemical properties of wood.
